# Helicobacter pylori related dyspepsia: prevalence and treatment outcomes at University Kebangsaan Malaysia-Primary Care Centre

**DOI:** 10.1186/1447-056X-8-4

**Published:** 2009-05-12

**Authors:** Aznida Firzah Abdul Aziz, Zuhra Hamzah, Seng Fah Tong, Sukumar Nadeson, Sharifa Ezat Wan Puteh

**Affiliations:** 1 Department of Family Medicine, Universiti Kebangsaan Malaysia Medical Centre, Jalan Yaacob Latiff, 56000 Kuala Lumpur, Malaysia; 2 Department of Surgery, Universiti Kebangsaan Malaysia Medical Centre, Jalan Yaacob Latiff, 56000 Kuala Lumpur, Malaysia; 3 Department of Community Health, Universiti Kebangsaan Malaysia Medical Centre, Jalan Yaacob Latiff, 56000 Kuala Lumpur, Malaysia

## Abstract

**Background:**

Optimum management of dyspepsia in primary care is a debatable subject. Testing for Helicobacter pylori (HP) has been recommended in primary care as this strategy will cure most underlying peptic ulcer disease and prevent future gastro duodenal disease.

**Methods:**

A total of 98 patients completed Modified Glasgow Dyspepsia Severity Score Questionnaire (MGDSSQ) at initial presentation before undergoing the ^13^Carbon Urea Breath Test (UBT) for HP. Those with positive UBT received Eradication Therapy with oral Omeprazole 20 mg twice daily, Clarithromycin 500 mg daily and Amoxycillin 500 mg twice daily for one week followed by Omeprazole to be completed for another 4 to 6 weeks. Those with negative UBT received empirical treatment with oral Omeprazole 20 mg twice daily for 4 to 6 weeks. Patients were assessed again using the MGDSSQ at the completion of treatment and one month after stopping treatment.

**Results:**

The prevalence of dyspepsia at Universiti Kebangsaan Malaysia-Primary Care Centre was 1.12% (124/11037), out of which 23.5% (23/98) was due to HP. Post treatment assessment in both HP (95.7%, 22/23) and non HP-related dyspepsia (86.7%, 65/75) groups showed complete or almost complete resolution of dyspepsia. Only about 4.3% (1/23) in the HP related dyspepsia and 13.3% (10/75) in the non HP group required endoscopy.

**Conclusion:**

The prevalence of dyspepsia due to HP in this primary care centre was 23.5%. Detection of HP related dyspepsia yielded good treatment outcomes (95.7%).

## Background

Dyspepsia is described as chronic or recurrent pain or discomfort in the upper abdomen. The prevalence of dyspepsia in western countries is approximately 25% which accounts for 2–5% of primary care consultations [1,2]. One of the challenges in treating dyspepsia for primary care physicians is to determine the optimal treatment for the patient presenting with new onset or previously uninvestigated dyspeptic symptoms [3].

Dyspepsia could be due to several causes such as peptic ulcer disease, reflux disease, drugs (especially Non-Steroidal Anti-Inflammatory Drugs, NSAIDs) and idiopathic. Symptoms commonly overlap, making diagnosis difficult. Gastro-oesophageal reflux disease (GORD) presents with predominant or frequent (more than once a week) heartburn or acid regurgitation [4]. Hence, it is often difficult to distinguish between dyspepsia and GORD in the uninvestigated patient who presents with upper gastrointestinal symptoms in primary care.

Almost one third of patients presenting with dyspepsia in primary care have peptic ulcer disease. In patients undergoing endoscopy for peptic ulcer disease, detection and eradication of Helicobacter pylori (HP) has demonstrated a potential cure for patients presenting with dyspepsia. Kurata and colleagues in their study noted that there is a significantly higher prevalence of peptic ulcer disease in dyspeptic patients who test positive for HP compared to those who do not [5]. Early endoscopy may be theoretically desirable for all patients but this is currently not practical [2]. Moreover, it is an invasive procedure, costly and causes discomfort to the patient.

Immediate referral for endoscopy is recommended for patients on regular NSAIDs and those with alarm symptoms such as weight loss, bleeding, anaemia, dysphagia, jaundice and palpable mass [6]. The recommendation of endoscopy in older patients is made because of concerns over the risk of underlying malignancy with increasing age [7].

To date, the main options for the treatment of younger patients with uninvestigated dyspepsia without alarming features include the following [7]:

(1) Empirical H_2_-receptor antagonist therapy;

(2) Empirical proton pump inhibitor (PPI) therapy

(3) HP testing and treatment of positive cases *(HP test and treat) *followed by acid suppression if the patient remains symptomatic

(4) Early endoscopy alone

(5) Early endoscopy with biopsy for HP and treatment if positive

(6) Acid suppression followed by endoscopy and biopsy if the patient returns symptomatic; or

(7) HP test and treat with endoscopy if the patient remains symptomatic

The Maastricht 2–2000 guidelines and primary care guidelines for the management of HP infection recommend a test-and-treat approach without endoscopy for adult patients under 45 years presenting in primary care with persistent dyspepsia [8,9].

However, there is much more limited evidence on the test and treat approach in Malaysia.

The United States National Institutes of Health (NIH) [10] recommends the carbon labelled urea breath test (UBT) as the best diagnostic approach because of its intrinsic operational advantages. Sensitivities of greater than 90% and specificities approaching 100% [10] make UBT the gold standard for diagnosis of active HP disease [8,11]. To prevent a false negative result, the UBT should not be administered within four weeks of proton pump inhibitor (PPI), bismuth or antibiotic therapy [12].

However, the test and treat approach is not currently practised at primary care level in Malaysia. Apart from private diagnostic laboratories, only Universiti Kebangsaan Malaysia Medical Centre (UKMMC) has this facility. This test is not available in any of the government-funded hospitals across Malaysia.

The management of dyspepsia is an important issue for both primary care physicians and specialists as the initial approach may dictate both patient outcome and future consumption of health care resources. This study aims to determine the prevalence of HP related dyspepsia and treatment outcomes among patients attending the UKM Primary Care Centre, a university funded primary care clinic. The extension of this service to the primary care facility from the UKMMC will facilitate the initiation of eradication at primary care level, hence limiting endoscopy for cases resistant to treatment. The study also aims to demonstrate the improvement of symptoms of dyspepsia in both groups of HP positive and negative patients after appropriate treatment.

## Methods

This cross-sectional study was conducted at UKM Primary Care Centre, a teaching primary care centre located in an urban area south of Kuala Lumpur. The study was conducted over a period of six months (January to June 2005). All new and 'follow-up' patients who attended UKM Primary Care Centre during the study period were screened for symptoms suggestive of dyspepsia and subsequently managed by the research team. Patients who were 18 to 50 years old and had symptoms of dyspepsia for at least four weeks were included in the study. Those excluded from the study were patients who had associated alarm symptoms (i.e. anaemia, dysphagia, gastrointestinal bleeding, jaundice, lymphadenopathy, palpable mass, significant weight loss), patients on NSAIDs, anticoagulant and steroid drugs, patients previously diagnosed, or had undergone surgery for gastroenterological or hepatobiliary disease, patients previously on eradication regimes for HP infection, patients with previously positive finding on endoscopy for other gastric pathologies, patients with family history of gastric cancer and patients who were pregnant.

The nature of the dyspepsia was assessed by determining the most predominant symptoms experienced by the patients. This involved a researcher interviewing the patients and asking them what they considered to be their most troublesome and frequent symptoms (i.e. epigastric pain, bloating, nausea, burping/belching, heartburn, sour taste or halitosis). The patients were then asked to fill a self-administered questionnaire regarding severity of dyspeptic symptoms using the Modified or Abbreviated Glasgow Dyspepsia Severity Score. Patients with language problems were referred to either a researcher or the paramedical staff who were pre-trained for the period of study.

The patients were then subjected to Urea Breath Test (GRAF Medical System) using ^13 ^carbon urea following the method described in the UBT study tool. Specimens were sent for analysis as soon as possible or not more than one week. Patients were reviewed and informed about the result and if the breath test result was positive, the patients received a regime of: oral Omeprazole 20 mg twice daily, Amoxycillin 500 mg twice daily & Clarithromycin 500 mg daily for one week

This regime was chosen as it has been proven to achieve consistent eradication of rates greater than 90%. (Based on Malaysian Academy of Medicine Consensus on Management of Peptic Ulcer Disease [13])

### Instruments

#### Modified Glasgow Dyspepsia Severity Score

This tool consists of an abbreviated version of the original Glasgow Dyspepsia Severity Dyspepsia Score [14], which has been shown to be a valid, responsive and reproducible means of assessing the severity of dyspepsia to allow measurement of symptoms over a one-month period.

#### ^13 ^Carbon Urea Breath Test (UBT)

A period of fasting before the test was not mandatory but timing in relation to the last meal was at least two hours before testing. Ingestion of carbonated drinks before the procedure was not allowed. Patients were instructed to take a deep breath and to count from 1 to 5 while blowing into the chamber bag slowly. Patients were given a drink containing the substrate (75 mg IRIS ^13^carbon urea, GRAF Medical Systems) and orange juice in a 3/4 glass of water. The patients were instructed to blow at indicated time intervals (10, 20, 30 minutes) into the respective chambers. The filled chambers were transported for analysis either immediately or within one week. UBT positive indicates HP-related dyspepsia while UBT negative indicates Non-HP-related dyspepsia

### Ethical approval

This study obtained ethical approval from the Medical Research Center, Faculty of Medicine, Universiti Kebangsaan Malaysia (#FF-179-2004).

### Data analysis

The data was analyzed using SPSS™ (Statistical Program for Social Sciences) software program version 11.5. Pearson's Chi square was used for detection of differences in categorical variables between the two groups (HP and Non HP). Mann Whitney's test was used for analysis of non-normally distributed data. The paired t-test was used to assess the mean scores pre and post treatment. The unpaired t -test used to assess the relation between quantitative and qualitative variables. All tests were done using a priori level of significance of 0.05.

### Sample size

Using the Epidinfo^® ^programme and a prevalence range of 30 to 40% at 95% confidence interval, the calculated sample size required was 92 patients.

## Results

Over the six month period of the study, a total of 124 patients presented with dyspepsia, making the prevalence of dyspepsia in adult patients attending PPP-HUKM 1.12% (124/11037). Further analysis on dyspeptic participants (98/124) recruited into this study, 23.5% (23/98) tested positive for urea breath test (HP-related dyspepsia) and 76.5% (75/98) tested negative for urea breath test (Non HP-related dyspepsia).

Both the HP and non HP-related dyspepsia groups showed statistically significant decline in the post treatment scores following treatment with eradication and empirical therapy respectively. (Table 1)

**Table 1:** Paired t-test in symptoms score decline pre and post treatment in HP and Non-HP related dyspepsia

		**Symptom score** **Mean (SD)**			
**Group**	**N**	**Pre Treatment**	**Post Treatment**	**Score Decline**	*95% Confidence Interval*
HP	23	8.43 ± 1.56	0.87 ± 1.01	7.57 ± 1.59	6.88 ± 8.25 *

Non HP	75	7.00 ± 1.52	1.05 ± 1.43	5.95 ± 1.92	**5.20 ± 6.39 **^†^

*p = 0.0008, ^†^p = 0.0008

Post eradication assessment of those with HP-related dyspepsia demonstrated 95.7% (22/23) complete or almost complete resolution of dyspepsia symptoms (score 0–1), whereas only 1 (4.3%) continued to experience dyspepsia (score more than 1). In the Non HP- related dyspepsia group, 13.3% (10/75) did not respond to treatment, with a mean score of 4.5. There were a higher proportion of patients in the non-HP group who required further endoscopy compared with the HP-related dyspepsia group (13.3% vs. 4.3%). (Table 2)

**Table 2:** Treatment outcomes in patients presenting with dyspepsia

	**ENDOSCOPY**	
	**No**	**Yes**
**HP**	95.7% (22/23)	4.3% (1/23)
**Non HP**	86.7% (65/75)	3.3% (10/75)

Total	88.8% (87/98)	11.2% (11/98)

## Discussion

Since HP was first cultured by Warren and Marshall in 1983, much has been learned about its clinical aspects and its epidemiology. Knowledge of the epidemiology of this infection comes mainly from prevalence studies. Investigation of the incidence of HP infection has been limited due to difficulties in identifying the case at the onset. In general HP infection is more frequent in developing countries than in developed nations. In developed countries, HP infection is acquired at fairly constant rate of 2–6% per year with prevalence 20–40% in adults [15]. Malaysian data shows that the prevalence of HP infection varies widely from 11 to 70% with an average of 35 to 40%. The highest rate were seen among the indigenous natives (54 to 65.3%) in east coast of Sabah, East Malaysia [16].

The overall prevalence of HP- related dyspepsia below the age of 50 years old found in this study was 22.5% (23/98). This is at the lower end of the range in the study by KL Goh where the overall prevalence of HP ranged from 26.4 to 55.0% in various parts of Malaysia, covering the Peninsular as well as East Malaysia [16].

The decline in the prevalence of HP infection seen in this study may reflect the steady improvement of our socioeconomic conditions resulting in decreased transmission as living conditions and hygiene improved [16]. Moreover, the setting for this study was done in an urban based primary care clinic with the majority of the population from the lower to upper middle income groups.

Our study also recorded the individual's predominant symptom to ensure that definition of dyspepsia, (which is pain or discomfort at centre upper abdomen), is strictly followed and to exclude patients who presented with heartburn (gastro-oesophageal reflux disease, GORD) as a predominant symptom.

This study has shown that the treatment response was good with most of the respondents achieved complete or almost complete resolution of dyspepsia symptoms. The use of symptomatic response to HP eradication therapy as a marker of post treatment status has been evaluated by McColl (1998) who concluded that complete resolution of dyspeptic symptoms is a powerful predictor of eradication of HP infection in ulcer patients. However, persistence of symptoms is a weak predictor of persisting infection; hence patients with persisting dyspepsia must have their HP status rechecked to guide further management [14]. This was supported in this study by the endoscopy findings of the participants who were referred for persistent symptoms. Eleven (11.2%) of them were referred; 10 (13.3%) were from the non HP-related dyspepsia group and only one (3.3%) from the HP-related dyspepsia group. Four of them did not turn up and the results of the other seven were traced.

Post treatment review of the HP and non HP related dyspeptic patients who had persistent symptoms, tissue biopsies taken via endoscopy tested negative for HP infection. Persistence of symptoms was found to be due to pan gastritis, reflux oesophagitis and mild gastritis.

Our study showed that a small number of patients required referral for endoscopy (1/23, 4.3%) when using the UBT as an initial approach in young patients with dyspepsia without alarm symptoms. This was similar to findings of other studies [2,8,10,11].

Although the test and treat strategy showed encouraging results, we do note that there are limitations to our study. The results obtained only reflected findings from a single urban based primary care centre. It does not reflect the overall population of primary care attendees which are largely based in the suburban and rural areas. Further multicentred studies looking into the cost effectiveness of testing for HP using the UBT should be done.

## Conclusion

Based on this study, the prevalence of HP related dyspepsia among adult patients attending a primary care centre is 23.5% using the UBT. The treatment response using eradication regime (with Omeprazole, Calrithromycin and Amoxycillin for one week) showed encouraging response with only 4.3% (1/23) requiring endoscopy.

UBT should be an option for detection of HP related dyspepsia in primary care setting based on its non invasive, high patient acceptance and highly sensitive features.

## Competing interests

The authors declare that they have no competing interests.

## Authors' contributions

AFAA participated in the design and coordination and drafted the manuscript.

ZH conceived the study, carried out the clinical trial, performed the statistical analysis and helped to draft the manuscript.

NS participated in the design, coordinated and performed the Endoscopy studies.

SEWP and TSF participated in the design of the study and statistical analysis.

All authors read and approved the final manuscript.
